# Consumer engagement in living evidence “a beautiful opportunity”: International qualitative study with patients and methodologists

**DOI:** 10.1002/gin2.70002

**Published:** 2024-09-13

**Authors:** Anneliese Synnot, Laura Weeks, Sophie J. Hill, Lyubov Lytvyn, Tamara Radar, Rebecca Randall, Richard Morley, Allison Jaure, Julian H. Elliott, Tari Turner

**Affiliations:** ^1^ Australian Living Evidence Collaboration, Cochrane Australia, School of Public Health and Preventive Medicine Monash University Melbourne Victoria Australia; ^2^ Centre for Health Communication and Participation, School of Psychology and Public Health La Trobe University Melbourne Victoria Australia; ^3^ Canadian Agency for Drugs and Technology in Health Ottawa Ontario Canada; ^4^ McMaster University Hamilton Ontario Canada; ^5^ MAGIC Evidence Ecosystem Foundation Oslo Norway; ^6^ Health Consumer Representative Canberra Australian Capital Territory Australia; ^7^ Cochrane London UK; ^8^ Sydney School of Public Health The University of Sydney Sydney New South Wales Australia; ^9^ Centre for Kidney Research The Children's Hospital at Westmead Westmead New South Wales Australia; ^10^ Future Evidence Foundation Melbourne Victoria Australia

**Keywords:** consumer engagement, living evidence, living guidelines, living health technology assessment, living systematic review, patient and public involvement

## Abstract

**Objective:**

We aimed to explore the opportunities, challenges and practical strategies for consumer engagement (i.e., patient and public involvement) in living evidence (systematic reviews, guidelines and health technology assessments that are continually updated with the latest evidence).

**Study Design and Setting:**

In this international qualitative study, methodologists (producers of systematic reviewers, guidelines and health technology assessments) with an interest in living evidence, and consumers (patients, informal carers, the public and their representatives) with experience contributing to evidence synthesis production participated in either a face‐to‐face workshop, online focus group or semistructured interview. We analysed data using descriptive synthesis.

**Results:**

Forty‐one methodologists and seven consumers from nine countries participated. A minority of participants in both groups had direct experience with living evidence synthesis. We identified seven themes: harnessing consumer enthusiasm in recruitment; ‘better’ consumer engagement based on deeper relationships; improved and ongoing orientation, support and remuneration; maintaining an ongoing commitment; potentially different guideline development stages and tasks; larger groups of consumers and multiple roles; and ongoing incorporation of consumer insights.

**Conclusion:**

Methodologists and consumers believe living evidence approaches present an imperative and an opportunity to explore new models of consumer engagement, bringing together larger and more diverse communities of consumers in true partnerships with methodologists. Consumer engagement strategies for living evidence allow ongoing improvement to engagement methods and ongoing incorporation of consumer experiences, preferences and values as they develop and change over time.

## Key points

*Key findings*

- Guideline methodologists and consumers believe living evidence presents an imperative and an opportunity to do consumer engagement ‘better’, envisaging a more ambitious model for consumer engagement.

*What this adds to what was known?*

- The living evidence concept may hold great appeal to consumers.

*What is the implication and what should change now?*

- A more ambitious consumer engagement model for living evidence would include a broader range of consumers engaged in a true partnership with methodologists, and each other, and well supported in an iterative approach that evolves and strengthens over time.

## 1 INTRODUCTION

Living evidence synthesis approaches (living systematic reviews, living guidelines and living health technology assessments) are a recent phenomenon, where evidence syntheses are continually updated to reflect new evidence as it emerges.^1^ By streamlining production processes, harnessing new technology such as machine learning, and working together in new ways, evidence producers can generate systematic reviews, guidelines and health technology assessments that retain their currency, relevance and validity.^2^ Living systematic reviews are now published by leading health and medical journals, such as Cochrane,^3^ The BMJ^4^ and Annals of Internal Medicine,^5^ while living guidelines are produced internationally by groups including the WHO,^6^ NICE^7^ and the Australian Living Evidence Collaboration.^8^, ^9^, ^10^ Living health technology assessments are at a more emergent stage.^11^, ^12^

There has been much consideration of the best methods and processes to realise the vision of living evidence.^1^, ^8^, ^11^, ^13^ Fundamental to these considerations is the fact that the core methodological steps underpinning traditional evidence production (e.g., comprehensive searches and transparent decision‐making) still apply. This includes actively involving patients, informal carers, the public and their representatives (‘consumers’) in the process to ensure that their views, experiences and preferences are reflected.^8^

Consumer engagement in systematic reviews, guidelines and health technology assessments has a long history.^14^, ^15^, ^16^ Consumers can be involved in all stages of the planning, conduct and dissemination of evidence production,^17^, ^18^, ^19^ with best practice principles and frameworks^20^, ^21^, ^22^ to support evidence producers to meaningfully involve consumers in ways that benefit the outputs. More broadly, the expectations around consumer engagement in research are changing, with an emphasis on respectful and inclusive partnerships between researchers and consumers (e.g., coproduction)^23^ and a desire to involve more people in new ways.^24^

The production of living evidence has numerous implications for how consumers might be best engaged and supported. All parties involved in living evidence must meet more frequently over a longer time and undertake time‐critical document reviews, discussion and decision making. Even in these early days, some living systematic reviews^25^ and living guidelines^8^, ^26^ are engaging consumers in their planning, conduct and dissemination. Two recent studies have explored consumers' and guideline methodologists' vision for consumer engagement in diabetes living guidelines^27^ and their practical experiences of consumer engagement in a series of living guidelines;^28^ however, important questions remain about the pros and cons of different consumer engagement approaches and how the findings apply beyond guidelines. The aim of this study was to explore the opportunities, challenges and practical strategies for consumer engagement in living evidence.

## 2 METHODS

This study was conceived in the course of early conceptual and methodological planning for living systematic reviews. Data collection occurred in 2018 and 2020, with analysis conducted in 2022–2023 (the delays were unintentional, for reasons unrelated to the project). Over this time, the interest and activity in living evidence have accelerated, with numerous examples of living systematic reviews and guidelines now published. Given the rapid uptake of living approaches during this period, consumer engagement in living evidence has moved from a hypothetical to an actual scenario over the course of this research, emphasising the importance of building and sharing knowledge and practice in this area.

Given the exploratory nature of the topic, we undertook a qualitative study, employing a general inductive approach within a pragmatic research paradigm.^29^ We also employed participatory methods, with an author team that included both researchers and consumers to oversee the project.^30^ Our reporting has been informed by the Standards for Reporting Qualitative Research (SRQR) checklist.^31^

### 2.1 Researcher characteristics and reflexivity

The mixed‐gender author team is from high‐income countries and includes systematic review, guideline and health technology assessment methodologists (Anneliese Synnot, Laura Weeks, Sophie Hill, Lyubov Lytvyn, Tamara Radar, Allison Jaure, Julian Elliott and Tari Turner), all of whom have practical experience with consumer engagement in evidence production. It also includes one consumer representative (Rebecca Randall) and one patient involvement coordinator (Richard Morley). The lead author (Anneliese Synnot) is a research expert in living evidence and consumer engagement. Some team members have been involved in the conceptualisation and methods development for living systematic reviews and living guidelines (Anneliese Synnot, Laura Weeks, Julian Elliott, Rebecca Randall and Tari Turner). All co‐authors have a deep commitment to consumer engagement in evidence production, and in health research, more broadly. The team has a positive bias towards living evidence and the value of consumer engagement, which may have influenced the analysis and themes generated.

### 2.2 Participants and recruitment

We included methodologists (producers of systematic reviewers, guidelines and health technology assessments) with an interest or experience in living evidence and consumers (patients, informal carers, their representatives and members of the public) with experience in evidence production. Given the novelty of the topic, we did not stipulate that consumers needed experience with living evidence. We recruited methodologists via email invitations through the Living Evidence Network (an international group of, at the time, over 200 evidence producers, publishers and users with an interest in living evidence), and member groups of the Australian Living Evidence Collaboration. We recruited consumers via email invitations sent by members of the project team (in the United Kingdom, Canada and Australia) to their consumer networks.

We conducted two rounds of recruitment (in 2018 and 2020) and included all participants who expressed an interest and could subsequently attend an interview, workshop or focus group. We did not recruit participants until data saturation was reached as data analysis did not commence until sometime after data collection was complete. We do, however, feel confident that the themes presented were consistent and repeated, and that after completion of data analysis, no new themes were emerging.

### 2.3 Data collection

After an initial 60‐min online focus group with five members of the author team (Laura Weeks, Sophie Hill, Lyubov Lytvyn, Tamara Radar and Rebecca Randall) to pilot our questions, we conducted a 30‐min face‐to‐face workshop with methodologists at a Living Evidence Network meeting in Scotland, UK in September 2018. In March–April 2020, another round of data collection was conducted with methodologists and consumers. Participants attended either a 60‐min online focus group (three focus groups conducted) or a 30‐min one‐on‐one interview (for those unable to attend a focus group; four interviews conducted). The methodologist and consumer focus groups and interviews were conducted separately.

At the start of each session, participants were given a brief presentation to introduce the living evidence concept (for consumers, we also provided written material on this topic beforehand). The sessions were led by experienced qualitative researchers (Tari Turner or Anneliese Synnot). We asked participants in the workshop, focus groups and interviews the following three questions:

- What comes to mind when you think about consumer engagement in living evidence?
- What should/could consumer engagement look like in a living evidence approach?
- What are the challenges and opportunities?

Detailed notes were taken for all sessions, with audio recordings made to supplement the written notes and provide deidentified illustrative quotes. Participants were not offered any remuneration for taking part.

We included the data from our pilot online focus group with the author team in the analysis. Such an approach is appropriate within research at the nexus of pragmatic (where researchers use the most appropriate methods to answer the question at hand)^29^ and participatory (where there can be considerable blurring of the boundaries between researchers and participants)^30^ inquiry. We did this as the topic was an emerging issue in which the global expertise was very limited, and all members of the project team also met the study inclusion criteria.

## 3 DATA ANALYSIS

The notes from all sessions were analysed using descriptive synthesis to identify key themes. In Microsoft Word, one author (Anneliese Synnot) applied inductive codes and grouped and refined the codes to form themes and subthemes, with review and revisions from a second coauthor (Tari Turner).

## 4 RESULTS

### 4.1 Participant characteristics

We included 48 participants; 41 methodologists and seven consumers. Methodologists included 36 Living Evidence Network members (from Australia, Canada, the United Kingdom, Germany, Chile, Lebanon, France and the United States), and five Australian living guideline developers. Consumers came from Australia, the United States, Canada and Israel. The consumers had experience producing one or more systematic reviews, health technology assessments or guidelines, with two having been involved in living guidelines.

We identified seven themes, described below, and listed in Box 1.

Box 1 **Seven themes describing the opportunities, challenges and practical strategies for consumer engagement in living evidence.**

1. Harnessing consumer enthusiasm in recruitment
2. ‘Better’ consumer engagement based on deeper relationships
3. Improved and ongoing orientation, support and remuneration
4. Larger groups of consumers and multiple roles
5. Potentially different guideline development stages and tasks
6. Maintaining an ongoing commitment
7. Ongoing incorporation of consumer insights

### 4.2 Theme 1: Harnessing consumer enthusiasm in recruitment

Most consumers described living evidence as an exciting and important concept, with consumers and methodologists predicting it was likely to have great appeal to consumers. They also suggested consumers may find the resulting evidence more relevant and trustworthy.

There is a lot of demand and interest amongst consumers. The [living] guidelines [I was involved in] were overwhelmed. [Consumer]

It's very confusing to read a systematic review that is a snapshot in time and if it's not current how do I know it's still relevant. It's much more relevant to consumers to have a living review. There's some kind of trust in that. [Consumer]

Consumers suggested that patient groups would be keen to be involved and that fostering relationships with these groups could provide ongoing access to experienced, well‐supported contributors, with a range of lived experiences.

They [patient groups] are connected—they will get their information out there. There is nobody more motivated to do living evidence than the patients in these groups. [Consumer]

It's harder to onboard someone into a living guideline process if they haven't participated in guidelines before. I feel that the more professional consumer representatives and groups are particularly helpful in this instance. [Methodologist]

Consumers and methodologists described existing challenges in finding consumers with the desired mix of lived, representative and/or evidence production experience. They wondered if the time commitment and perceived higher level of expertise required of consumers contributing to living evidence might exacerbate these issues.

If you go with only the patient participants who can devote a lot of time you really narrow the body of participants, and you don't get a broader perspective of people. [Consumer]

If the topic is an area in which there isn't an experienced consumer community, we may need to provide consumers with more support, and we might have trouble finding people. [Methodologist]

### 4.3 Theme 2: ‘Better’ consumer engagement based on deeper relationships

Consumers and methodologists described that living evidence presents both an imperative and an opportunity to get consumer engagement ‘right’. Participants felt that the imperative to involve consumers related to what they saw as the wide appeal of living evidence to consumers and the fact that living evidence production seemed like an inevitable future.

Consumer engagement in (…) systematic reviews is important but in living evidence there's an even greater case for it. I think there would be a lot more confidence for the general public if they knew that it was living evidence. It's important to get it right. [Consumer]

We are writing the rules with the living evidence [approach] therefore it is a unique opportunity to change the rules and bring consumers into the foreground without the baggage of an entrenched process. [Methodologist]

Similarly, developing good relationships between consumers and methodologists was seen as both imperative to the success of consumer engagement in living evidence, and a wonderful opportunity inherent in the living evidence model.

A good partnership/relationship between the patients and the researchers. You need to have an open line to the researcher because if you need to be updated often you need the straight line to the editor or author. [Consumer]

Because the projects are long‐term there is a beautiful opportunity for team building, to find consumers strengths, to develop relationships. It's all about relationships. [Consumer]

Methodologists also highlighted that living evidence means the consumer engagement approach can be more dynamic.

[There is an] opportunity to be a lot more agile and to test different processes. Nothing is set in stone, we can test different models of consumer engagement even within the same guideline, but it's not so risky. [Methodologist]

Some methodologists suggested that the practice of consumer engagement in living evidence may not be that different compared with traditional evidence production. However, many participants outlined the ways in which consumer engagement in living evidence could differ, as articulated in the following themes.

### 4.4 Theme 3: Improved and ongoing orientation, support and remuneration

Consumers highlighted that unclear expectations, insufficient orientation and lack of payment are common experiences when they have been involved in traditional evidence production. They highlighted the importance of adequate support for consumers embarking on a long‐term commitment. Two consumers outlined that contributing to living evidence could be more ‘difficult’ or ‘challenging’ for consumers, highlighting the importance of adequate orientation to this new concept and ensuring support is available on an ongoing basis.

What was good about [being involved in developing a rapid recommendation] was that I had a really clear person to go to, and they gave you really good training. These things could happen in a living evidence approach. [Consumer]

For those of us who are asked to participate and give up hours of time to be offered no money (or just expense reimbursement) is an insult. Patients need to be paid, particularly as the effort may be ongoing. [Consumer]

Participants agreed that due to the nature of living evidence production, consumer engagement activities will need to be predominantly online. Consumers experienced with living evidence highlighted the loss of the informal support they previously enjoyed when attending face‐to‐face meetings with other consumers, and the need to offer technical support.

The thing that I'm missing now on the living guideline is having the contact with other people to chat about stuff. Sometimes when you chat about stuff you get the connection. [Consumer]

Participants also identified that methodologists will need guidance and resources to ensure they can adequately support consumers, including training, financial resources and time.

[The need for] funding is heightened in a living evidence world. To do consumer engagement properly requires an upfront investment and funding to keep it going. [Methodologist]

### 4.5 Theme 4: Larger groups of consumers and multiple roles

Participants felt that including a larger group of consumers, in the form of a network or panel, would allow methodologists to include a broader range of experiences and perspectives, and provide a way for inexperienced consumers to contribute while meeting the need for timely response to tasks, and mitigating the loss of consumers over time.

When you go out and do focus groups you meet so many patients and carers who want to be involved but don't know how. This will provide an opportunity for them to be involved. [Methodologist]

With living, (…) it's how you engage with consumers that doesn't tire them out. How do we ensure we have the depth and timeliness of input, so there is ability to reach out when we need to but they're not overloaded? [Methodologist]

Consumers and methodologists suggested living evidence needed a multi‐layered consumer engagement model, where consumers with different experiences and skill sets could contribute at different levels (e.g., governance vs. project level) and to different topics and tasks. Some methodologists experienced with living guidelines advocated including a paid consumer member of the living evidence team.

If not constrained by resources, I imagine a layered consumer engagement model, that is, 1–2 consumer researchers, who are paid members of the research group plus a citizen‐based group (potentially online) who can jump in and out of specific tasks as needed, and as they are able. [Consumer]

### 4.6 Theme 5: Potentially different living evidence stages and tasks

Some participants suggested it may be important to prioritise consumers' input into particular stages of living evidence production (e.g., defining the questions), with several participants suggesting an increased role for consumers in the dissemination and implementation of living evidence.

Consumers would be heavily engaged in guiding formats for the outputs of living evidence—could even be involved in writing text of guidelines and other evidence products. [Methodologist]

Consumer engagement is most useful when defining and prioritizing [living systematic review] questions. [Methodologist]

Others described the importance of engaging with consumers early and throughout the process.

For living guidelines, I see consumers as being fundamental for [all guideline development stages, but they're] embedded in the teams as lived experts, so they bring a different perspective and work hand in hand [with guideline developers]. It's about weaving the consumers into the journey. [Methodologist]

Consumers and methodologists suggested there might be new tasks for consumers, specific to living evidence, including contributing to decisions about which topics are suitable for living evidence, thresholds for when to update or ‘retire’ a topic, and considering the impact of any new evidence.

If you take a review that's already been published and you want to convert it into an LSR [living systematic review], that's the time to ask consumers if they've asked the right question. [Consumers]

### 4.7 Theme 6: Maintaining an ongoing commitment

Methodologists wondered whether consumers may find it challenging to contribute for an undefined period of time and whether their motivation would wane over time. This concern was not voiced by consumers.

It's quite a change from being involved in a defined period [where it's] all done in a year and then you have a break. For us, it's our job, but for a consumer, doing it voluntary, or with some fees, it's a change in commitment in time and availability. [Methodologist]

One methodologist suggested that maintaining an adequate number of consumers and providing ongoing support could present a challenge for methodologists.

Repeated recruitment, orientation (…), the need to account/manage drop outs and how to bring in new people over time, that is, ongoing recruitment, selection, retention and support. [Methodologist]

### 4.8 Theme 7: Ongoing incorporation of consumer insights

Participants also envisioned that living approaches could allow for continual incorporation of patient experiences, preferences and values data over time. This might mean including more and different consumers' perspectives, and/or updating the guideline to reflect consumers' experiences as they develop or change over time (e.g., with newly available treatments).

Great opportunity for incorporating otherwise unheard insights that have not been tapped before as patients begin to have experience with the new drugs, there is an opportunity to include that in the ongoing health technology assessment. [Consumer]

Are there ways we can consider gathering patient‐reported outcomes and patient experience in real‐time data to feed into the guidelines? A possible future. [Methodologist]

## 5 DISCUSSION

In this exploratory study, we found that both guideline methodologists and consumers believe living evidence presents an imperative and an opportunity to do consumer engagement ‘better’. They envisaged a model for consumer engagement that is underpinned by partnerships with patient groups, whereby larger groups of consumers are recruited to different roles and/or tasks across a living evidence project. Such an approach would allow a broader range of perspectives while mitigating potential challenges related to timelines and attrition over time. Participants shared differing views about whether consumers should be involved in priority evidence production stages (e.g., dissemination and implementation) or involved throughout. In previous research, a multilayered model of engagement (involving a consumer think tank, guideline panel members and ‘crowd’) was proposed for diabetes living guidelines^27^ while our previous work^28^ has shown involving larger groups of consumers in iterative and evaluated engagement activities is well suited to living guidelines.

In designing strategies for consumer engagement in living evidence work, participants urged that attention be paid to commonly overlooked aspects of consumer engagement, such as establishing clear expectations, orientation, remuneration and support for consumers, along with guidance and resources for methodologists. They identified potential challenges related to finding and selecting consumers with the necessary mix of experience, skills and availability. The importance of doing engagement ‘well’ and paying particular attention to recruitment and selection were both highlighted in the two recent studies on consumer engagement in living evidence.^27^, ^28^ The main differences between consumer engagement in living evidence production and conventional evidence production include the need to support an ongoing commitment from consumers, potentially recruiting more people and the possibility of consumers contributing to new tasks specific to living evidence. Broadly speaking though, good practice in consumer engagement in living evidence production builds on established principles of good consumer engagement in conventional evidence production.^21^, ^22^

A limitation of this study is the fact that data was collected before the rapid scaling up of living evidence precipitated by the COVID‐19 pandemic. This meant most participants had limited or no experience with living evidence. Their insights would undoubtedly have been richer and possibly, different, if data collection occurred, or had re‐occurred, in 2024. We also acknowledge our sample included only a small number of consumers and that many of the participating methodologists (particularly those associated with ALEC and the author team) had long histories of involving consumers in their guideline programmes and viewed consumer engagement favourably. However, the similarities between their views and those of consumers and methodologists with direct experience of living evidence^28^ support the prescience of our findings. An additional limitation relates to the limited diversity of participants. Most were from high‐income countries (with none from Africa) and we spoke to a few people who did not belong to the majority cultural group within their country, which represents an equity issue in who was able to contribute to this research. Given the differences in guideline and health technology assessment development in high‐ versus low‐ and middle‐income countries,^32^, ^33^ and the additional considerations for involving people from marginalised groups in evidence production,^34^ our findings may have limited applicability to these contexts.

In terms of implications for practice, an important finding of this study is the desire of methodologists and consumers to build a better and more ambitious model of consumer engagement in living evidence. Such a model would include a broader range of consumers engaged in a true partnership with methodologists, and each other, and well supported in an iterative approach that evolves and strengthens over time. Such models would likely also have great relevance to conventional evidence production. The rapid uptake of living evidence internationally demonstrates that methodologists see the value of living evidence and our results suggest this interest might be shared by consumers. Although we only spoke to a small number of consumers, their enthusiasm for the concept of living evidence suggests finding interested consumers may not be difficult, but methodologists will need considerable expertise and sufficient time and resources to select the right people, offer flexible approaches and ensure consumers are well supported and remunerated. While living approaches were essentially designed to allow systematic reviews and guidelines to be updated as evidence changes, they should also be updated as consumers' experiences, preferences and values change over time. We need further research that is co‐designed with consumers to build and test different consumer engagement models to continue to develop guidance for consumer engagement that is specific to living evidence.^8^

## 6 CONCLUSIONS

Methodologists and consumers believe living approaches present an imperative and an opportunity to explore new models of consumer engagement, bringing together larger and more diverse communities of consumers in true partnerships with methodologists. The development of strategies for consumer engagement in living evidence synthesis presents an opportunity to address known challenges to consumer engagement, continue to refine and improve engagement methods and allow ongoing incorporation of consumer experiences, preferences and values as they develop and change over time.

## AUTHOR CONTRIBUTIONS

**Anneliese Synnot**: Conceptualisation; investigation; methodology; formal analysis; writing—original draft; writing—review and editing. **Laura Weeks**: Conceptualisation; methodology; investigation; writing—review and editing. **Sophie J. Hill**: Conceptualisation; methodology; writing—review and editing. **Lyubov Lytvyn**: Conceptualisation; investigation; methodology; resources; writing—review and editing. **Tamara Radar**: Conceptualisation; methodology; resources; writing—review and editing. **Rebecca Randall**: Conceptualisation; investigation; methodology; writing—review and editing. **Richard Morley**: Conceptualisation; methodology; resources; writing—review and editing. **Allison Jaure**: Methodology; writing—review and editing. **Julian H. Elliott**: Conceptualisation; investigation; methodology; writing—review and editing. **Tari Turner**: Conceptualisation; investigation; methodology; writing—review and editing; supervision.

## CONFLICT OF INTEREST STATEMENT

AS is member of the Editorial Board of Clinical and Public Health Guidelines, but she did not participate in the editorial process of this paper and had no inﬂuence on the editorial decisions related to it. The other authors declare no conflict of interest.

## ETHICS STATEMENT

All participants provided informed consent. Ethical approval was obtained from the Monash University Human Research Ethics Committee (no. 14831) with reciprocal approval from the La Trobe University Science Health and Engineering College Human Ethics Sub‐Committee.

## Supporting information

Supporting information.

Supporting information.

## Data Availability

We have included excerpts of the qualitative data collected as illustrative quotes within the manuscript. Additional data have not been uploaded to a data repository and cannot be shared publicly as consent was not sought from participants to do so.
