# Supplementary material for: Consumer engagement in living evidence “a beautiful opportunity”: International qualitative study with patients and methodologists
Source: Clin Public Health Guidel. 2024 Sep 13;1(4):e70002. doi: 10.1002/gin2.70002 (PMC13563385; doi:10.1002/gin2.70002)
Supplement: Supplementary file 1 [file GIN2-1-e70002-s001.docx]

**INVITATION EMAIL FOR CONSUMERS**

Hello,

My name’s Annie Synnot and I work at Cochrane Australia, Monash University in Melbourne, Australia. You are receiving this email as I am keen to talk to people who have experience bringing a consumer/patient/citizen perspective to the development of systematic reviews, clinical guidelines or health technology assessments, as part of a study I am involved in.

**What are we researching, and why?**

Together with a group of international colleagues, I am exploring the potential opportunities and challenges of consumer engagement (also known as ‘patient and public involvement’) in a new model for producing systematic reviews, clinical guidelines and health technology assessments. This new model is called ‘living evidence’ (see short explainer of this concept at the bottom of this email).

To do this, we are inviting people with a range of experiences in the production of evidence to join us in an online discussion group. Your views and ideas will be used to inform how we embed consumer/patient engagement within our living evidence model, before we test it in future pilots.

The project has been approved by Monash University’s Ethics Committee, and more detail is attached in the Explanatory Statement.

**Who would we like to hear from?**

People who have experience bringing a consumer (or patient/public) perspective to systematic reviews, guidelines or health technology assessments.

Note: You do not need to be familiar with the concept of living evidence to take part!

**What will you be asked to do?**

What: Join between 4 to 7 other people in a 60-minute online group discussion with myself and our expert facilitator. We will first explain what we mean by ‘living evidence’ then ask the group a series of questions about your views and ideas. We will also send you this explanation of ‘living evidence’, and our planned questions before the session, so you have time to ‘digest’ it all before the meeting.

When: We would like to hold these discussion groups in November/December. We will probably have 2 or 3 different meeting times, so we can accommodate all the international time differences.

How: You will be able to join via your computer, or dial in via a local telephone number.

**What next?**

If you have questions, please feel free to email me at xxxxx

If you are happy to take part, please send me an email me at [xxxxx](mailto:Anneliese.synnot@monash.edu) and let me know where you live (city/country) and your usual availability (i.e. if there are particular days of the week, or times of the day that are preferred or in which you are unavailable).

Kind regards,

Annie Synnot

*’Living evidence’ describes a new way of producing evidence that allows near real-time updating of systematic reviews, health technology assessments, evidence-based guideline recommendations, and patient decision aids. We plan to do this by using the latest developments in technology, such as artificial intelligence and online tools, and also by forming stronger and potentially larger partnerships with all relevant stakeholders, including consumers/patients. The ultimate aim of living evidence is to shorten the time it takes for new research knowledge to influence health policy and practice, thereby improving patient outcomes.

**INVITATION EMAIL FOR EVIDENCE PRODUCERS AND METHODOLOGISTS**

Hello,

The living evidence team at Cochrane Australia, along with a group of international colleagues, are currently conducting a project exploring the potential opportunities and challenges of consumer engagement (also known as patient and public involvement) in living evidence. To do this, they are inviting people with a range of experiences in systematic reviews or guidelines to join them in an online discussion group.

**Who would they like to hear from?**

Evidence producers or methodologists who are:

- Experienced in systematic reviews, guidelines or health technology assessments AND
- Have either experience, or a high level of understanding of, the concept and activities involved in living evidence.

Ideally, these participants will also have an interest or experience in engaging consumers in the planning, conduct and/or dissemination of systematic reviews, guidelines or health technology assessments

**What will you be asked to do?**

Join about 10 other people in an online group discussion with the research team. This will be held at a time that is most convenient for what is likely to be an international group of participants.

The session will go for 90 minutes and will be led by an expert facilitator who will ask the group a series of questions about your views on this topic. Before the session, we will send you some background reading (about 20 minutes’ worth) introducing the concepts of living evidence and consumer engagement.

**Want to find out more, or express your interest in participating?**

Please email Annie Synnot at Cochrane Australia at [Anneliese.synnot@monash.edu](mailto:Anneliese.synnot@monash.edu) with any questions, or to express your interest in participating.
